# Detection of American Football Head Impacts Using Biomechanical Features and Support Vector Machine Classification

**DOI:** 10.1038/s41598-017-17864-3

**Published:** 2017-12-21

**Authors:** Lyndia C. Wu, Calvin Kuo, Jesus Loza, Mehmet Kurt, Kaveh Laksari, Livia Z. Yanez, Daniel Senif, Scott C. Anderson, Logan E. Miller, Jillian E. Urban, Joel D. Stitzel, David B. Camarillo

**Affiliations:** 1 Stanford University, Stanford, CA USA; 2 Stevens Institute of Technology, Hoboken, NJ USA; 3 University of Arizona, Tucson, AZ USA; 4 Wake Forest University, Winston-Salem, NC USA

## Abstract

Accumulation of head impacts may contribute to acute and long-term brain trauma. Wearable sensors can measure impact exposure, yet current sensors do not have validated impact detection methods for accurate exposure monitoring. Here we demonstrate a head impact detection method that can be implemented on a wearable sensor for detecting field football head impacts. Our method incorporates a support vector machine classifier that uses biomechanical features from the time domain and frequency domain, as well as model predictions of head-neck motions. The classifier was trained and validated using instrumented mouthguard data from collegiate football games and practices, with ground truth data labels established from video review. We found that low frequency power spectral density and wavelet transform features (10~30 Hz) were the best performing features. From forward feature selection, fewer than ten features optimized classifier performance, achieving 87.2% sensitivity and 93.2% precision in cross-validation on the collegiate dataset (n = 387), and over 90% sensitivity and precision on an independent youth dataset (n = 32). Accurate head impact detection is essential for studying and monitoring head impact exposure on the field, and the approach in the current paper may help to improve impact detection performance on wearable sensors.

## Introduction

Accumulation of head impacts may contribute to acute and long term brain trauma. In rodent models, repeated head impacts can worsen brain injury outcomes and substantially prolong recovery^1–3^. In football players, sustaining a large number of high severity impacts over an athletic event is associated with elevated concussion risk^4^, and repeated injuries led to increased vulnerability to subsequent injury^5^. Even without clinically diagnosed concussions, players with a greater number of head impacts in a game showed higher levels of blood brain barrier disruption than those with fewer impacts^6^. At lower impact severities and exposure levels, a bout of soccer headers led to acute neurological deficits and concussion symptoms^7,8^. Aside from acute injuries, head impacts over a season of football has been shown to lead to white matter integrity changes^9^. In fact, long-term head impact exposure from sports and other activities may trigger a neurodegenerative condition called chronic traumatic encephalopathy^10,11^. With heightened awareness of the association between sports head impacts and brain trauma, guidelines have been introduced to limit head impact exposure in sports, such as the recent US Soccer organization’s recommendation of no heading for young children^12^. In response to such concern, many head impact sensors have been developed to measure head impact exposure.

Some sensors use simple linear acceleration thresholds to differentiate head impacts from normal motions. Although commonly used, this algorithm is likely insufficient. When a low acceleration threshold is set for impact detection, large numbers of false positives are expected, since inertial sensors can pick up accelerations from many sources including sensor handling. On the other hand, if a high acceleration threshold is used, some true impacts are likely missed, while high acceleration false positives will remain. Realizing that acceleration thresholding alone may be inadequate, some companies have developed proprietary algorithms for impact detection, but there is little published validation of their performance, and the limited amount of validation data reveal problems with these approaches.

Current impact detection methods have problems with false negative and false positive detections. In a Head Impact Telemetry System (HITS) validation paper, 19% of dummy impacts were incorrectly removed by its impact detection algorithm^13^. In cadaver drop tests, the HITS detected 79.6% of drops, G-Force Tracker (GFT) detected 87.0%, skin-adhered patches mounted behind the left ear and right ear detected 86.1% and 75.7%, respectively^14^. Considering that lab dummy impacts are relatively simplified scenarios and isolated events, more complex field conditions may lead to even more false negatives in these sensors. For false positive detection, a field validation study of the skin-mounted xPatch sensor found more than 30% of the recordings to be spurious^15^. Another study using video analysis to verify sensor impacts found that 65% could be verified for the G-Force Tracker and only 32% could be verified for the xPatch sensor^16^. Many of these sensors with impact detection problems have been deployed to sports players to report impact exposure and study injury mechanisms^17–20^. Without accurate impact detection, sensor data may include substantial amounts of false positive and false negative readings, which can skew injury mechanism findings, and lead to inconsistent and inconclusive results.

Due to the complexity of human motion and activities, detection of such events with wearable sensors has benefitted from more complex features and algorithms. Aside from commonly used time-domain linear acceleration features, features may be extracted from angular motion and from the frequency-domain transformations of the time-domain signals. In fact, since the temporal dynamics of human motion are often distinguishable from those of other activities, Fourier Transform features (frequency-domain) and Wavelet Transform (WT) features (time and frequency-domain) have proven useful in detecting falls and other human activities^21–25^. In addition, some studies for detecting human motion also used knowledge-based features to impose biomechanical feasibility constraints for detection. For example, in a fall detection paper, the authors describe the validation of a constraint based on the fact that human thigh segments normally do not tilt beyond a certain threshold except during falls^26^. These features combined with machine learning algorithms such as support vector machines (SVMs) have yielded high performance in past research^27–30^. Therefore, employing more features from the time and frequency domains of kinematics as well as machine learning algorithms may be necessary to generate a high-performance classifier.

In detecting head impacts, one study used a neural network algorithm combined with discrete Fourier transform heuristics to distinguish between head impacts and nonimpacts in soccer^31^. Raw time-domain data were used as features in the neural network classifier, and the classifier was evaluated to have 88% sensitivity and 47% specificity. In pilot work^32^, we have demonstrated efficacy of power spectral density (PSD) features combined with an SVM classifier in differentiating dummy head impacts and laboratory-simulated nonimpacts with 98% sensitivity and 99.98% precision. Although this approach showed promise in the laboratory setting, field conditions are expected to be more complex and may require additional features and refined techniques. In the current work, our objective was to identify the distinguishing features of collegiate football head impacts in the field, and use these features to develop and validate a head impact classifier.

## Methods

**Figure 1:**
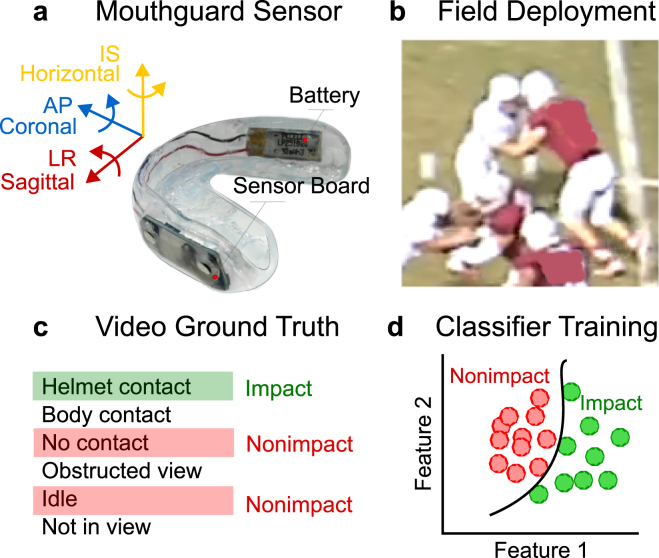
Study Overview. We deployed instrumented mouthguards (**a**) to football players during games and practices (**b**). Videos of the events were recorded to generate ground truth event labels for mouthguard recordings (**c**). Using the labelled dataset containing head impacts and nonimpact events, we trained a classifier to distinguish between these two classes of events (**d**).

We deployed instrumented mouthguard sensors to collegiate football players during regular season games and practices to gather a ground truth dataset of head impacts and nonimpacts to train and validate a head impact classifier (Fig. 1). In the training and validation set, we included video-verified helmet contact recordings as head impacts, and video-verified noncontact recordings as nonimpacts. Prior to training the classifier, we used infrared (IR) device placement measurements to filter out recordings where the mouthguard was not coupled to the upper jaw. Then, features were extracted from the kinematic sensor measurements to train an SVM classifier that distinguishes between impacts and nonimpacts. To validate the performance of the classifier, we used leave-one-out cross-validation. In addition, we tested the classifier trained and validated using collegiate football data (n = 387) on a small independent youth football dataset (n = 32). The training and testing data supporting this article have been uploaded as part of the supplemental material.

### Sensor Deployment and Field Data Collection

We deployed instrumented mouthguards^32,33^ to 7 collegiate football players (1 center, 1 tight end, 1 guard, 1 running back, 1 fullback, 1 wide receiver, and 1 linebacker) over 32 player-events (including 12 player-games and 20 player-practices) at Stanford University and 3 youth football players (1 center, 1 cornerback, and 1 linebacker) over 6 player-events (3 player-games and 3 player-practices) at Wake Forest University. The collegiate football games followed the 2015 National Collegiate Athletic Association (NCAA) Football Rules, and the youth football games followed the 2015 American Youth Cheer (AYC) League Football Rules. The practices were typically 1–2 hours long. The collegiate football players and youth players wore the Riddell Speed Helmet. The number of players and events were chosen to acquire a similarly sized dataset as the previous laboratory impact detection study using dummy head impacts and human subject reconstructed nonimpacts (n_impact_ = 128, n_nonimpact_hiIR_ = 118)^32^. Human subject protocols at each site were respectively approved by the Stanford Administrative Panels for the Protection of Human Subjects and the Wake Forest Reynolda Campus Institutional Review Board, with all data collection conducted in accordance with the institutional review boards’ guidelines and regulations, and informed consent was obtained from each participant. The mouthguard couples to the skull via the upper dentition to measure skull motion, and has approximately 10% error in measuring peak head linear acceleration, angular acceleration, and angular velocity in dummy head validation^33^. We recorded full 6-degree-of-freedom linear acceleration and angular velocity (anterior-posterior or AP, left-right or LR, inferior-superior or IS linear accelerations, and coronal, sagittal, horizontal plane angular velocities^34,35^) of the skull at 1000 Hz sampling rate for 100 ms (10 ms pre-trigger and 90 ms post-trigger with 10 g trigger). In addition, an IR sensor detected the presence of teeth within the mouthguard tray at the time of impact to determine when the mouthguard was taken off teeth.

### Video Analysis

Practice and game videos were analyzed in detail to identify ground truth activity labels for the data recordings on the mouthguard. Since the application of interest was head impact detection, we were most interested in distinguishing data recorded during head impacts (helmet impacts in the case of football) from other mouthguard recordings (e.g. running, jumping, mouthguard manipulation without contact). The system was not trained on head acceleration activity without direct head contact (e.g. where body contact could lead to head acceleration without direct head impact), since it is difficult to judge and label such activities from video. Body contacts that may have involved head accelerations were excluded from the head impact classifier training for this study. Video methods are detailed in a separate publication^36^, but briefly outlined below. For the collegiate football dataset used for training and validation, during each game, 1080 p 30 fps videos were taken from two views: one from the sideline and one from the end zone, such that each play would be captured by both videos. During a practice, 1080 p 30 fps videos were taken from four views of the practice field: typically one from each end zone/sideline, such that each practice activity was captured by at least one video. To achieve sufficient resolution in recording contact activity, camera operators moved the camera views to focus the field of view on contact activity. In a first round of video analysis, evaluators labeled player activity throughout an event for each video angle independently. Each time segment in the video was labeled with a specific category of player activity (e.g. 15:50:36.12–15:50:36.15, helmet contact). The categories of activities labeled were helmet contact (player’s helmet observed to be impacted by any object), body contact (player’s body observed to be impacted by any object without helmet contact), no contact (player is in physical activity but not in contact with any person or object), idle (player observed to stand by), obstructed view (player is in activity but view of their helmet is obstructed), and no view (player is not in video view). In this round of video review, evaluators were instructed to have high sensitivity in identifying any period when the helmet may have contacted another object, and the inter-rater reliability for helmet contact identification was found to be 88%. In a second round of video analysis, an author (LCW) reviewed all labeled helmet contact periods by cross-referencing all video angles. At this stage, any helmet contact periods when the contact was questionable or verified to not occur in a different video angle were excluded from the ground truth set. In addition, the directionality (frontal/facemask, left, right, rear, or top of helmet being impacted, which is similar to commonly used football impact directionality definitions^37^ with additional distinction of facemask impacts), type of contact (helmet or facemask to helmet or ground or body), and qualitative severity of the contact were noted for reference. The independent youth football event videos had a single video angle, but a human operator redirected the camera view to follow practice activities. A similar video analysis procedure was performed on the youth data to establish helmet contact and noncontact labels. Without multiple video angles, only data recordings where contact could be confidently identified in the single video view (e.g. lateral view of a head-on collision) were included in the dataset.

### Ground Truth Training and Validation Dataset

The ground truth labels on the mouthguard data were established by cross-referencing the video analysis, which was conducted independent of the mouthguard data. As mentioned in the last section, the first round of video analysis provided activity labels throughout the durations of the practices and games, and the second round of video analysis specifically verified potential helmet contact periods. Using this information, we identified recordings in mouthguard data that fall within the helmet contact periods, as well as in periods where no contact was observed. To minimize noise in the training dataset, we imposed strict selection criteria for recordings to include in the ground truth labelled dataset. The general approach was to match video observations with mouthguard recordings as closely as possible, such that selected helmet contact recordings have a high likelihood of resulting from helmet contacts seen in video, and nonimpacts have low likelihood of including any helmet contact recordings. Thus, in the training dataset, we excluded any data recordings for which we had insufficient information to judge their labels, such as the recordings during no view periods.

To extract the ground truth impact (helmet contact) dataset, we performed three steps. First, we synchronized real timestamps on the mouthguards and the game/practice videos. After synchronization, we cross-referenced video timestamps and mouthguard data timestamps to extract the labeled ground truth mouthguard dataset. Since the mouthguard timestamp had a resolution of 1 second, and video-mouthguard synchronization could also have at least ±1 second error, we used a ±2 second time window to select potential helmet contact periods from the mouthguard data, i.e. any mouthguard recording occurring within 2 seconds of a video-identified helmet contact period was selected. Second, to further ensure that the mouthguard helmet contact recordings matched with video-observed helmet contacts, an additional helmet impact directionality constraint was imposed where we eliminated impacts for which neither the peak acceleration direction nor the integrated head motion direction matched with the video-observed impact directionality. Third, we eliminated recordings where there was higher power in the high frequency domain (above 200 Hz) than the low frequency domain (below 200 Hz), since such recordings have a high likelihood of resulting from high frequency mouthguard electronics noise, and with a 1000 Hz sampling rate, such high frequency dynamics would be under-sampled even if they resulted from helmet contact. To extract the ground truth nonimpact dataset, we included recordings within the time windows with either the no contact label or the idle label, such that the mouthguard recordings likely resulted from noncontact activities (e.g. running) or sideline activities (e.g. mouthguard manipulation).

### Infrared Device Placement Classification

First, we extracted the IR sensor reading to determine sensor placement. The IR sensor in the mouthguard detected the presence of teeth within the mouthguard tray, and could help filter out recordings where the device was not properly placed on the upper dentition to record skull kinematics. The IR readings for on-teeth and off-teeth recordings typically exhibit bimodal distributions, and thresholds for determining device placement were set to separate the two distributions^32^. Thresholds were determined on a per-subject-event basis (game or practice) because of individual variabilities in the mouthguard and wear and tear over time. Because we assumed a bimodal distribution of IR readings representing off-teeth low readings and on-teeth high readings, we used MATLAB’s fitgmdist function to fit a dual Gaussian to the IR data collected over a subject-event. The threshold was then determined as the IR reading at which the high on-teeth Gaussian and the low off-teeth Gaussian cross. We eliminated any impact or nonimpact recordings from the training set with low IR, since if the IR reading was lower than the threshold, the mouthguard may have been off-teeth or loosely coupled.

### Feature Extraction and Analysis

For feature extraction, raw kinematic signals gathered from the mouthguard (triaxial linear acceleration and angular velocity time series) were rotated to the head anatomical frame, but the linear accelerations were not transformed to the centre of gravity of the head, to avoid cross-coupling of linear and angular features. For each subject, the rotation matrix for transformation was determined based on individual measurements of the orientation angles of the sensor board with respect to the anatomical planes, with reference to the dental model. The rotated linear acceleration and angular velocity time series were filtered according to each sensor’s bandwidth (accelerometer cutoff – 500 Hz, gyroscope cutoff – 184 Hz) using a fourth order Butterworth filter. Then, the linear velocity time series in each anatomical axis was calculated by integrating the linear acceleration in each axis over the 100 ms duration of the impact using MATLAB’s cumtrapz function. The angular acceleration time series in each anatomical axis was calculated by differentiating the angular velocity in each axis over the 100 ms duration of the impact using the five point stencil method^38^. The vector magnitudes of linear acceleration, linear velocity, angular acceleration, and angular velocity were also calculated. The kinematics signals were used to extract the following features. In total, we extracted 411 features (see Supplemental Table for descriptions of all features).

#### Time-Domain Features

Maxima of linear acceleration, change in linear velocity, angular acceleration, and change in angular velocity were extracted in each anatomically aligned directional component and in vector magnitude. Impulse durations of linear acceleration and angular acceleration were extracted by calculating the width of the impulse at half the maximum value.

#### Power-Spectral Density Features

Power spectral density (PSD) amplitude of linear acceleration and angular acceleration were extracted in each directional component and in vector magnitude. We extracted linear acceleration PSD values and angular acceleration PSD values at frequencies from 10 Hz–200 Hz and 10 Hz–180 Hz, respectively, in intervals of 10 Hz. As mentioned, the gyroscope has a bandwidth of 184 Hz, which is why the 180 Hz upper bound is chosen for the angular signals. According to Society of Automotive Engineers (SAE) J211 guidelines^39^, sample rate is suggested to be six times the signal bandwidth. With a sample rate of 1000 Hz, we expected that the linear acceleration signals above approximately 200 Hz to be undersampled, and chose an upper bound of 200 Hz for the feature extraction.

#### Wavelet Transform Features

Wavelet Transform (WT) of a signal is the representation of the signal in real space as a linear combination of wavelet basis functions, which are localized in both frequency and time. The main advantage of WT is that it provides not only the power spectral density at different frequencies but also presents quantitative information about their time dependence. Morlet WT was performed on the linear acceleration and angular acceleration signals in each anatomically aligned directional component and vector magnitude. For each of these, the frequency, value, and timing relative to the time-domain peak of the peak WT amplitude were extracted. In addition, we estimated the bandwidth of the WT by calculating the frequency at which the peak WT amplitude dropped below 10% of the maximum value. Similar to the PSD features, we extracted peak WT amplitudes over time of linear acceleration and angular acceleration in each directional component and in vector magnitude at frequencies from 10 Hz–200 Hz and 10 Hz–180 Hz, respectively, in intervals of 10 Hz.

#### Biomechanical Modeling Features

We estimated kinematics of the neck and torso based on a 2-linkage biomechanical model of the head-neck-torso. The kinematic model comprises a head rigid body attached to a neck rigid body via a two-rotational-degree-of-freedom joint (universal joint). The axes of rotation for the universal joint are the left/right axis (representing the atlanto-occipital joint) and the inferior/superior axis (representing the atlanto-axial joint). The neck rigid body is further constrained such that its base (representing its attachment to the torso) has a minimal distance with its position at time t = 0. This minimization of distance constraint represents our assumption of a massive torso that has less motion than the less massive head. From this, we could define the orientation of the neck with three degrees of freedom rotation. Then, given we have six degrees of freedom knowledge of the head position (integrated from linear acceleration and angular velocity measurements from the mouthguard), we could solve for the universal joint angles at the head-neck joint, the ball joint angles of the neck, and the distance between the current position of the torso end and the initial position of the torso end. Thus, we had a fully determined system with six knowns (head degrees of freedom) and six unknowns (two head-neck angles, three neck angles, and one minimum distance constraint). For the model, we used a neck length of 10.8 cm^40^, and a head centre-of-gravity location of 5 cm above the head-neck joint^41^. From this model, we extracted several features. First, we extracted the percent time over the 100 ms duration that the angles between the head and neck linkages matched signs with the angles between the torso and neck linkages. This represented a continuous positive or negative rotation of the cervical spine. Next, we extracted the peak absolute joint angles, torso, neck, and head displacements (as measured at the base of the neck, base of the head, and centre of mass of the head respectively), and respective velocities and accelerations as a representation of the total motion of our model. Although the features extracted from the kinematics signals used untransformed linear accelerations to avoid cross-coupling of linear and angular features, the model-based features used linear accelerations transformed to the centre of mass of the head to estimate head velocities and displacements. Finally, we extracted ratios of linear and angular acceleration kinematics, which represented the biomechanical coupling due to the presence of the neck. Specifically, we took the ratios between the angular acceleration about each anatomical axis with respect to linear acceleration along the remaining two normal anatomical directions. The ratio was computed for each time sample in each impact, and the mean, standard deviation, and peak were all utilized as features.

### Two-Class Classifier Training and Validation

A two-class SVM classifier was trained to differentiate head impacts from the nonimpacts. The off-teeth recordings were not included in the SVM classifier training, since in practice we would be directly rejecting them using the IR feature. We trained an SVM classifier with a radial basis function kernel using the MATLAB fitcsvm function (Mathworks, Natick, MA).

First, sequential feature selection was used to prune the feature set to avoid overfitting (i.e. when the number of training examples is small compared to the number of fitting parameters, such that the fit is good for the training data but is not generalizable) and optimize classifier performance, using the MATLAB sequentialfs function. The feature selection used cost functions to optimize the classifier for (1) area under the receiver operating characteristics (ROC) curve (AUC), and (2) the F-measure, which is the harmonic mean of sensitivity and precision. These cost functions calculated AUC and F-measure from leave-one-out cross validation, instead of calculating the measures from training, such that the feature selection was optimized to avoid over-fitting. In the leave-one-out process, we trained the classifier with all but one sample from the full dataset (including impacts and nonimpacts), then used the trained classifier to classify the remaining sample, and aggregated the results from all possible combinations to calculate the cost function (i.e. number of cycles equals size of training/validation dataset). Leave-one-out cross validation maximized the number of data samples used for training (which is often desired when training data collection is resource-intensive), and best approximated how the classifier trained on all available data will perform given a newly acquired collegiate data sample.

Second, after feature selection, the leave-one-out cross validation performance of the AUC-optimized and F-measure-optimized classifiers was used to compare the two classifiers, specifically using sensitivity, specificity, accuracy, precision, area under the ROC curve, and area under the precision-recall (PR) curve as the performance criteria. To compare the performance of the optimized classifiers against a basic method, we also plotted the ROC and PR curves for a simple acceleration thresholding method, where the peak linear acceleration vector magnitude was used to classify between impacts and nonimpacts. To do this, we varied the classification threshold between the minimum and maximum peak linear acceleration vector magnitudes in the dataset to obtain the sensitivity-specificity or sensitivity-precision pairs for plotting the ROC and PR curves. The equations for sensitivity, specificity, accuracy, precision, and F-measure are included below (Equations 1–5).

1 $$Sensitivity=\,\frac{TP}{TP+FN}$$

2 $$precision\,=\,\frac{TP}{TP+FP}$$

3 $$Specificity=\,\frac{TN}{TN+FP}$$

4 $$Accuracy=\,\frac{TP+TN}{TP+FP+TN+FN}$$

5 $$F-measure=\frac{2\ast sensitivity\ast precision}{sensitivity+precision}$$

where true positive (TP) was a head impact that was classified by the classifier as a head impact, true negative (TN) was a nonimpact that was classified by the classifier as a nonimpact, a false positive (FP) was a nonimpact that was classified by the classifier as a head impact, and a false negative (FN) was a head impact that was classified by the classifier as a nonimpact.

### Further Evaluations of the Two-Class Classifier

After training and validation of the two-class classifier using collegiate football data, we also tested the trained collegiate two-class classifier on the small independent youth football dataset. Similar procedures as the collegiate data procedures were used to extract the test dataset from the youth data, and we evaluated sensitivity, specificity, accuracy, precision, and areas under the ROC and PR curves as the performance criteria. In addition, since the classifier was only trained on a small fraction of the mouthguard recordings that could be verified and labelled through video analysis, we performed qualitative evaluation of the classifier’s performance in classifying all data recordings for one player over an event in the collegiate dataset. For this evaluation, we used IR filtering and the trained classifier (optimized for AUC) to classify all data recorded during a single practice for an offensive guard with more than 1000 mouthguard recordings, and cross-referenced activity periods independently identified in the first round of video analysis (including helmet contact, body contact, and obstructed possible contact periods) with the classifier-identified helmet contact time points in mouthguard data. We call this a qualitative evaluation, since insufficient information is available from video to provide definitive activity labels for all mouthguard recordings to calculate performance measures. This evaluation mainly served to provide information on what proportion of mouthguard recordings may be classified as impacts and when these classified impacts occurred relative to independent video observations.

### Statistical Tests

To identify potential distinguishing features between impacts and nonimpacts, and considering that features were not necessarily normally distributed, we performed a two-sided unpaired Wilcoxon Rank-Sum test (also known as Mann-Whitney U-test) between the impact and nonimpact distributions of each feature, and multiple comparisons were corrected using the Bonferroni correction method to judge statistical significance (411 comparisons, with adjusted p-value threshold of 0.0001). Since there were a larger number of features compared to the number of training cases, we anticipated redundancy in the feature set and performed principal component analysis (PCA) on the standardized (zero mean, unit variance) feature matrix as well as Pearson’s correlation analysis between pairs of features.

## Results

### Ground Truth Impacts and Nonimpacts

**Figure 2:**
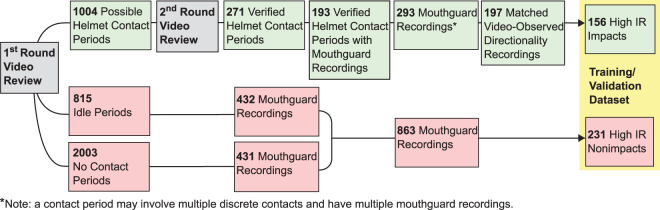
Extracted Training and Validation Dataset. In the first round of video review, raters identified periods of possible helmet contact, player being idle, or in activity without contact. To extract the impact set, a second round of video review was performed to establish confidence on the helmet contact label. Instrumented mouthguard recordings within verified helmet contact periods were further matched with video observed directionality and checked for device placement through infrared sensing before inclusion into the impact dataset. High IR mouthguard recordings from idle and no contact periods were included in the nonimpact dataset.

**Figure 3:**
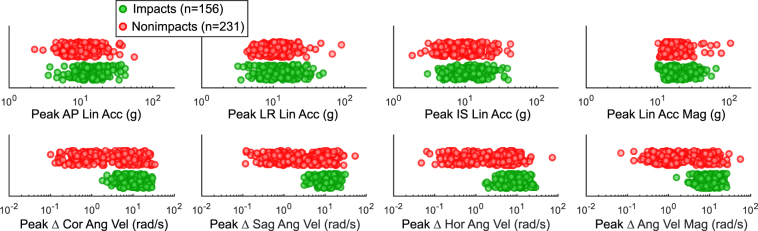
Distributions of Kinematics Measures for Impacts and Nonimpacts. Shown here are high IR impact (n = 156) and high IR nonimpact (n = 231) distributions of common kinematic measures that could be used for impact registration or classification. The subplots are anterior-posterior (AP), left-right (LR), inferior-superior (IS) linear acceleration, and coronal (Cor), sagittal (Sag), horizontal (Hor) plane angular velocity. It is shown that impacts and nonimpacts have similar linear acceleration distributions. Comparing linear acceleration magnitude, impacts had values ranging from 10.1 g to 65.6 g (mean 22.0 g, median 19.1 g), while nonimpacts ranged from 10.0 g to 104.3 g (mean 18.5 g, median 15.8 g). For change in angular velocity magnitude, impacts had values ranging from 1.6 rad/s to 26.1 rad/s (mean 10.5 rad/s, median 9.7 rad/s), while nonimpacts ranged from 0.1 rad/s to 55.7 rad/s (mean 5.4 rad/s, median 3.3 rad/s). Although there are more low-angular velocity recordings in nonimpacts compared to impacts, there are overlaps between the two classes. With the overlaps in distributions, the kinematics features may not be the most predictive features for classification.

As shown in Fig. 2, the two rounds of video review helped to identify periods of helmet contact, player being idle, or in activity without contact, to provide labelled impact and nonimpact data recordings for the training and validation dataset. Infrared sensing helped to exclude any recordings where the mouthguard may have had loose coupling from the teeth. In the ground truth impact and nonimpact dataset, there were 156 impacts and 231 nonimpacts. Features extracted from the kinematics data were used to classify the impacts and nonimpacts. The peak of linear acceleration vector magnitude is often used as a main feature for impact classification. In addition, angular velocity is a direct measurement from the gyroscope and may serve as an angular feature for impact detection. Figure 3 shows the distributions of linear acceleration and angular velocity peak kinematics for impacts and nonimpacts. The distributions of impact and nonimpact peak linear acceleration features overlapped. Even though more nonimpacts had low angular velocities, there was still overlap between the two classes.

**Figure 4:**
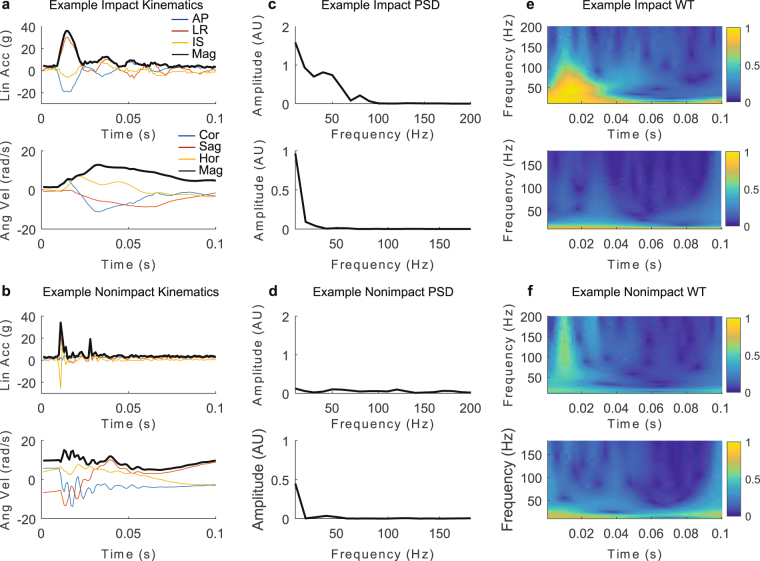
Kinematics, PSD, and WT of an Example Impact and Nonimpact. Example impact (**a**) and nonimpact (**b**) kinematics show qualitative differences between these two events, with the nonimpact exhibiting higher frequency impulses and oscillations. Such frequency-domain differences are reflected in the Fourier transform power spectral density (PSD) plots (**c** and **d**) and wavelet transform (WT) plots (**e** and **f**), where color represents amplitude.

One set of typical impact and nonimpact signals are shown in Fig. 4. Despite having similar peak kinematics in linear acceleration and angular velocity, the impact (Fig. 4a) and nonimpact kinematics (Fig. 4b) show qualitative differences. Nonimpacts tend to exhibit sharper impulses in linear acceleration and higher frequency oscillations in angular velocity compared to impacts. Comparing PSD amplitudes of the kinematic magnitudes (Fig. 4c and d), the impact had higher power at lower frequency ranges below 100 Hz for linear acceleration and below 50 Hz for angular velocity compared to the nonimpact. Comparing WT of the kinematic magnitudes (Fig. 4e and f), the impact had high linear acceleration WT amplitude within 100 Hz in the first 40 ms of the impact corresponding to the impact impulse. The angular velocity WT was low frequency (<50 Hz) throughout the impact. On the other hand, for the nonimpact, linear acceleration WT had high frequency content above 100 Hz for brief time segments, and angular velocity WT showed greater amplitudes in higher frequencies over the impact.

### Distinguishing Features and Feature Redundancy

**Figure 5:**
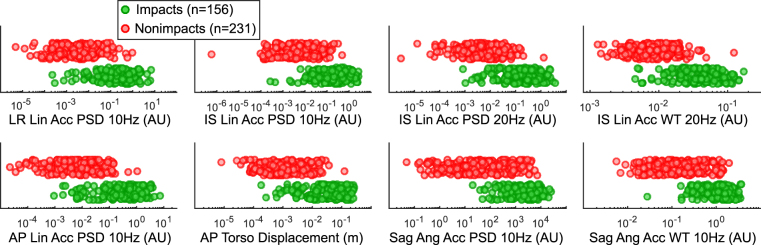
Distinguishing Features between Impacts and Nonimpacts. Here we show distributions of 8 features with the lowest p-value in the Wilcoxon Rank-Sum test between impacts and nonimpacts. These features include linear acceleration and angular acceleration PSD and WT features at low frequencies (10–20 Hz), as well as estimated AP torso displacement from the kinematic measurements. With less overlap between impacts and nonimpacts in these distributions, the features tend to have higher predictive value in distinguishing the two classes.

From the Wilcoxon Rank-Sum test, common peak kinematic features did not show as much distinction between impacts and nonimpacts compared to some frequency-domain features. Distributions of the eight features with the lowest p-values with significant differences between impact and nonimpact distributions (p ≪ 0.0001) are shown in Fig. 5. Most of these features are linear acceleration or angular acceleration PSD/WT features at low frequencies (10 or 20 Hz). Corresponding to the observations from Fig. 4c–f, these features show that impacts have higher PSD or WT amplitudes at low frequencies compared to nonimpacts, in all three anatomical directions for linear acceleration, and in the sagittal plane for angular acceleration. In addition, impacts also tend to have higher estimated torso displacement compared to nonimpacts.

**Figure 6:**
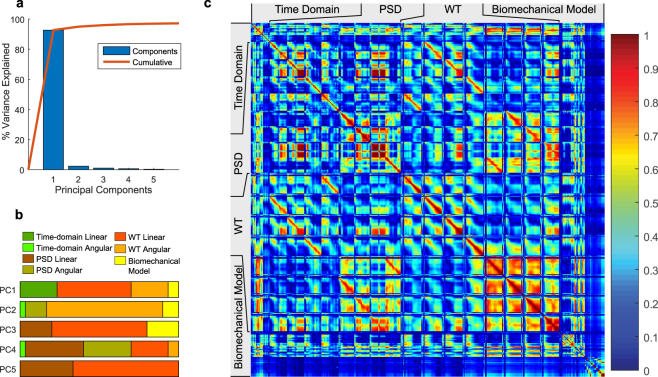
PCA and Correlation Analysis of Features. Principal component analysis of the feature space shows that 90% of the variance could be explained by the first principal component, indicating low dimensionality of the feature space (**a**). Among the first five principal components, the top contributing features are mainly PSD and WT features, and the time-domain linear acceleration features have a relatively significant contribution only in the first principal component (**b**). Pearson’s correlation analyses of features show that many features are highly correlated (**c**).

To check the dimensionality of the feature space and correlations among features, we performed PCA to show that the first principal component could explain over 90% of the variance in the feature matrix (Fig. 6a). An analysis of the top 30 contributing features of the first five principal components showed that most of the contributions came from PSD and WT features (Fig. 6b). The top contributing features of the first principal component were linear time-domain features, linear WT features, angular WT features, and biomechanical modelling features. Pearson’s correlation analyses between pairs of features showed that many features were highly correlated (Fig. 6c). In the time-domain (Supplemental Fig. 1), angular peak kinematics features tended to have higher correlations between each other. Among PSD features (Supplemental Fig. 2), the AP and LR linear acceleration features at neighboring frequencies were highly correlated with each other, and these were also highly correlated with angular velocity PSD features in the sagittal and horizontal planes. The angular acceleration WT features tended to be highly correlated with each other (Supplemental Fig. 3). In addition, the linear acceleration PSD features had high correlation coefficients with linear acceleration WT features, and similar observations were made for the angular PSD and WT features (Supplemental Fig. 4). Among the biomechanics modelling features, the model-based kinematics features tended to be correlated with each other (Supplemental Fig. 5).

### Best Performing Features and Classifier Performance

**Figure 7:**
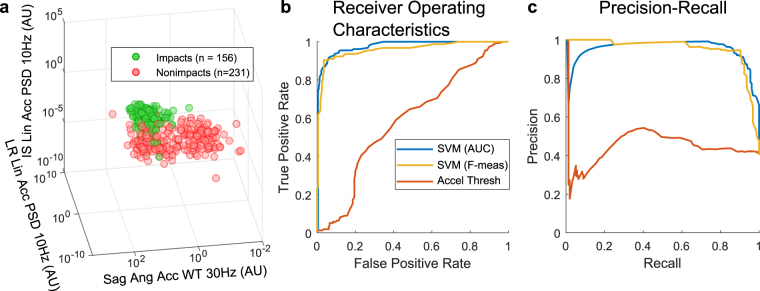
Best Performing Features and Classification Decision Boundary. The top three features from forward feature selection optimizing for AUC are shown in (**a**) with distributions of impacts and nonimpacts in the space of these three features. The ROC and PR curves of the AUC classifier and F-measure classifier show areas under curves of close to 1, while a classifier based on acceleration thresholding has similar performance as random guessing (**b**,**c**).

From forward feature selection, the best set of features to optimize the area under the ROC curve was (in order of feature selection): LR linear acceleration PSD at 10 Hz, sagittal plane angular acceleration WT at 30 Hz, IS linear acceleration PSD at 10 Hz, sagittal plane angular acceleration PSD at 70 Hz, linear acceleration magnitude WT at 20 Hz, and standard deviation of the ratio between linear acceleration and angular acceleration magnitudes over time. The distributions of impacts and nonimpacts in the space of the first three chosen features are shown in Fig. 7a. When optimizing for the F-measure, different features were selected: IS linear acceleration WT at 20 Hz, angular acceleration magnitude WT at 10 Hz, LR linear acceleration impulse duration, the ratio between IS linear acceleration and sagittal plane angular acceleration, horizontal plane angular acceleration PSD at 130 Hz, IS linear acceleration WT at 110 Hz, and horizontal plane angular acceleration PSD at 140 Hz.

We evaluated the AUC-optimized classifier and F-measure-optimized classifier using the cross validation performance measures, including sensitivity, specificity, accuracy, precision, and areas under the ROC and PR curves. The performance measures are shown in Table 1. These performance measures are calculated from the SVM classification of impacts and nonimpacts after IR filtering. The ROC and PR curves for the AUC-optimized and F-measure-optimized classifiers are shown in Fig. 7b and c. Both the classifier optimized for AUC (blue) and that optimized for the F-measure (yellow) had areas under the ROC and PR curves over 0.9 (AUC_ROC_ = 0.96 for AUC optimization, AUC_PR_ = 0.95 for AUC optimization, AUC_ROC_ = 0.98 for F-measure optimization, AUC_PR_ = 0.94 for F-measure optimization). On the other hand, a simple classifier just based on linear acceleration magnitude thresholding (red) did not perform much better than randomly assigning classes with a probability of impact of 50% (with area under the ROC curve close to 0.5). The classifier optimized for AUC had better ROC performance while that optimized for the F-measure had better PR performance. The classifier optimizing for the F-measure had higher performance measures than that optimizing for AUC, with all performance measures over 90%.

**Table 1:** Classifier Performance. Performance measures for the classifier were calculated using leave-one-out cross validation (cross validation columns). In addition, the classifier was tested on a small youth dataset and the same performance measures were reported (test columns).

	Optimized for AUC		Optimized for F1	
	*Collegiate Cross Validation*	*Youth Test*	*Collegiate Cross Validation*	*Youth Test*
Dataset Size	387	32	387	32
TP	136	15	141	15
TN	221	15	223	11
FP	10	1	8	5
FN	20	1	15	1
Sensitivity	87.20%	93.80%	90.40%	93.80%
Specificity	95.70%	93.80%	96.50%	68.50%
Accuracy	92.20%	93.80%	94.10%	81.30%
Precision	93.20%	93.80%	94.60%	75.00%
AUC_ROC_	0.96	—	0.98	—
AUC_PR_	0.95	—	0.94	—

**Figure 8:**
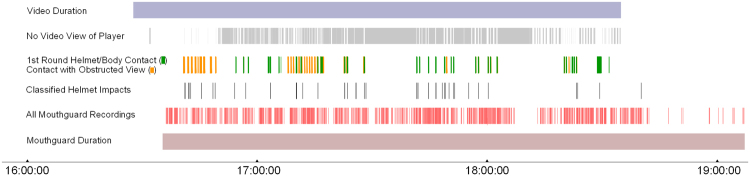
Classifier Performance in Classifying All Events Recorded During a Practice. Since the classifier was only trained on a small fraction of data that could be verified through video, we tested the performance of the classifier in classifying all events recorded by the mouthguard over a sample event (total recordings = 1219). This figure shows the timeline of a practice for an offensive lineman. Over the recorded video duration, the first round of video analysis identified periods of helmet or body contact, as well as when contact likely occurred but the type of contact could not be confidently judged due to obstructed video view. 46 events recorded by the mouthguard were classified as helmet contact by the trained classifier, and they tend to fall close to periods of observed or obstructed contact from video analysis.

The independent youth test dataset included 16 head impacts and 16 nonimpacts. We applied both trained classifiers on this independent test dataset, and the test results are shown in Table 1. Although the classifier optimizing for the F-measure had better cross-validation performance on the training and validation dataset, the classifier optimizing for AUC had higher performance measures on the test dataset, with over 90% performance for all measures. When we used the trained binary classifier to classify all data recorded (n = 1219) during a practice for a college offensive lineman, 46 of the 1219 data recordings were classified as head impacts, and the remaining recordings were either filtered out using IR (n = 1026) or classified as nonimpacts (n = 147). Shown in Fig. 8 are time points of classified head impacts compared with observations from video analysis. Over the recorded video duration, we show video-identified periods where helmet or body contacts were observed and periods where contacts may have occurred but view of the player’s head was obstructed. Most of the classified head impacts fell within or close to periods of observed or possible contact based on video analysis.

## Discussion

Here we present a study where we trained and validated a head impact classifier with collegiate field data. We combined the use of a head impact sensor with independent video analysis to provide training and validation data from collegiate football for the head impact classifier. Using this methodology, we have identified features that were helpful in distinguishing between helmeted football head impacts and nonimpacts on the collegiate football field.

### Classifier Performance

In our collegiate field data, the majority of the data recordings had low IR readings (73% of the nonimpacts and 23% of the recordings during helmet contact periods were low IR, or off-teeth), indicating that the mouthguard was likely removed from the teeth during these recordings. Since football is a unique sport where not all players are always in action, device removal could occur frequently especially when the player is idle and not in play. Since the amount of play time also varies from player to player and from event to event, the number of off-teeth recordings could vary accordingly. Thus, the IR sensing mechanism is helpful to easily reject such off-teeth recordings. For our application, the proximity sensing method needs to be specific for detecting whether the device is mounted on teeth, instead of just detecting the device being in mouth. Extending to other wearable head impact sensors, we advise that sensors include a suitable proximity sensing mechanism to sense device placement and reject recordings when the device is not properly mounted.

The SVM classifier helped to reject high-IR nonimpacts. Such recordings could occur when the player bites or chews the mouthguard while it is worn, or when other objects may be present in the mouthguard tray to generate a high IR reading (e.g. lip or finger). We trained two SVM classifiers using our collegiate football data, with one optimizing for area under the ROC curve and the other optimizing for the F-measure. For both optimization schemes, the first features chosen during the forward feature selection process were low-frequency PSD or WT features (LR linear acceleration PSD at 10 Hz for AUC and IS linear acceleration WT at 20 Hz for F-measure). Optimization for AUC is often used for problems where ranking of class probabilities is important and has been shown to be a superior metric than accuracy^42^, while optimization for the F-measure is usually helpful for detecting the rare class in unbalanced data^43^. In our data, after filtering with the IR signal, the numbers of impacts and nonimpacts are approximately balanced, in which case the F-measure may be less critical. On the other hand, optimizing for AUC may help to minimize the overlap of classes at the decision boundary in the transformed data space. In our study, the F-measure classifier had better cross-validation performance on the training data, while the AUC classifier had better and more consistent performance on the test data. This indicates that the F-measure classifier likely overfit to the collegiate football training and validation data. The AUC classifier had consistent performance on the collegiate training dataset as well as the independent youth test dataset. Based on this limited dataset, retraining of the classifier for youth football may not be necessary. To evaluate this assumption, future studies using a larger youth dataset that includes all positions and more extensive video data are warranted.

The rigorous video verification of ground truth labels helped to minimize noise in the ground truth training data. As a result, we only trained the classifier using a small fraction of all recorded data that could be verified and labelled through video analysis. Recognizing that, we also performed a qualitative evaluation of the classifier’s ability to classify all data recordings, as shown in Fig. 8. The training data for the classifier only incorporated about 10% of the data from this event. As expected, most of the recordings had a low IR reading (84%), and only a small proportion of the recordings (4%) among the 1219 recordings were classified as helmet impacts, which mostly fell close to video-identified periods of possible contact. Some helmet contact recordings identified with the classifier were in periods with obstructed view of the player’s helmet during contact, indicating that the impact detection algorithm may help to more sensitively identify helmet contact when the player’s action is obstructed in video.

### Distinguishing Features

Traditional peak kinematics features often used for impact detection could not distinguish between head impacts and nonimpacts as well as frequency-domain features. As seen in Fig. 3, the peak kinematics values largely overlapped between impact and nonimpact distributions, resulting in low predictive value for head impact detection, as confirmed by the red ROC and PR curves in Fig. 7. Even from qualitative inspection, it was clear that nonimpacts tended to have higher frequency impulses and oscillations compared to impacts. Such differences in frequency content were reflected in the PSD and WT features, and impacts had higher power at lower frequencies compared to nonimpacts. As a result, the PSD and WT features at low frequencies were most distinguishing between impacts and nonimpacts. In fact, the first three selected features to optimize ROC were PSD features at 10 Hz and a WT feature at 30 Hz which span all three anatomical planes of motion (Fig. 7a). This is likely because during most of the nonimpacts, the mouthguard may have been moving on its own. With much lower inertia, it would have higher frequency dynamics during such motion with lower power. On the other hand, head impacts involve more massive collisions between players and may thus exhibit lower frequency dynamics with higher power. WT features could pick up transient frequency modes that occur for short durations, such as the 30 Hz feature shown in Fig. 7a, which may be characteristic of transient helmet dynamics at the moment of impact.

From the principal component analysis and correlation analysis, it was shown that the feature space was highly redundant and most of the variance in the feature space could be explained by a small number of features. This redundancy is also illustrated by the fact that a small number of features were selected from the forward feature selection process to optimize performance. Increasing the number of features caused overfitting. However, due to the highly collinear feature space, the feature selection process may select different but correlated features due to slight differences in feature distribution that influence performance. This may also cause over-fitting, which may be mitigated through using a larger training and validation dataset.

### Limitations and Future Work

The classifier is only evaluated to be used with the specific instrumented mouthguard design used in this study for classifying collegiate American football helmet impacts. Retraining is likely required for any device that does not have the same mechanical and electrical design as this instrumented mouthguard and for detecting head impacts in other contexts. Although features of helmet impacts recorded on different head impact sensors may be similar, provided that these sensors can accurately measure skull motion, the kinematics of nonimpact recordings will likely differ for different device form factors and sensor locations. It is also possible that different helmet materials may lead to different head impact kinematics. In unhelmeted sports or other sports where different forms of contact and/or player behaviours are involved, impact and nonimpact characteristics are expected to change, requiring retraining and validation of the classifier. In addition, the current classifier was trained using data from 7 collegiate players. This dataset did not include data from all possible positions in American football, with one defensive player, a linebacker, and six offensive players. Prior to implementing the impact detection system for field use independent of video confirmation, testing of the classifier using an independent collegiate dataset from different players and positions will help further validate field performance. With the small youth dataset, we showed consistent performance of the classifier, but further validation with a larger youth dataset is likely required for validating the classifier in the youth population.

Using video information to establish ground truth data labels has some limitations. In this study, the impact and nonimpact dataset was rigorously selected to ensure that we had high confidence in the ground truth labels. This may have limited the classifier to clearly visible activities. For example, almost all recordings from when the player was buried under a pile were excluded from training, since a clear view of the player’s head/helmet could not be established. However, it is highly likely that helmet contact will occur during such periods and it may exhibit unique dynamics due to multiple impacts and simultaneous impacts to different objects (body, helmet, ground). In our evaluation, classifying all recordings in a practice showed that the classifier picked up some potential helmet contact recordings during periods of obstructed view. In addition, even with the 2–4 camera angles in the collegiate data collection, activity labels, especially contact labels, could not always be confidently determined due to factors including viewing angle, resolution, and distance. With the youth data collection, only one video angle was available due to field study restrictions, and as a result fewer activities could be confidently labeled with a clear view in the single video angle. Furthermore, video analysis does not provide kinematics information to judge which contact activities should have passed the recording threshold. Future studies may include more camera views with higher resolution and larger field of view to capture all activities on the field and provide more video information for judging event labels. Additional sensors and close-up cameras, such as helmet sensors and helmet mounted cameras, may provide additional kinematics and video information.

The instrumented mouthguard device was susceptible to mandible noise if the lower jaw was not clenched^44^. Although we have developed a design to isolate this noise^44^, the devices in the current study were of an older design^32^, thus the kinematic signals from some impacts may be coupled with high-frequency jaw noise. In the context of head impact detection, although high kinematic accuracy of sensors is not required, such noise may make it more difficult to differentiate between impacts and nonimpacts. Updated designs to eliminate jaw noise (such as Kuo 2016^44^) should further enhance classification performance. Also, the mouthguard in this paper had inertial sensors of limited bandwidth with additional filtering. It is possible that some nonimpacts may have high frequency content not captured by the sensors. If higher bandwidth sensors are used, and higher frequency PSD or WT features could be extracted, it is possible that the additional features may further distinguish between the two classes. For example, the current gyroscope bandwidth is only 184 Hz. Increasing this would help capture potential high frequency dynamics in nonimpacts. Similarly, lengthening the duration of the recording may capture lower frequency features (<10 Hz) that may be characteristic of slower body/neck dynamics. However, with increased recording time, multiple impacts may also be captured within the same recording, which is not investigated in the current study and may complicate classification.

While the IR mechanism was helpful in determining device placement and rejecting recordings where the device is not properly mounted, device designs to optimize coupling and subject compliance are still required to ensure sensitivity in detecting all helmet contact. For example, if the mouthguard is not coupled to the teeth during head impacts, even though the impact detection algorithm may be able to reject such recordings, it is not ideal since we fail to capture the head impact data. The current study uses an earlier version of the Stanford instrumented mouthguard, which was not optimized for subject comfort and coupling. Among all video-identified helmet contacts in the training data, about 71% were captured on the mouthguard, and only around 77% of the mouthguard recordings had high IR readings. Of the unrecorded impacts, 81% were observed to be light severity or involved body or facemask impacts and may not have triggered the 10 g linear acceleration threshold. It is also possible that some head impacts may have been missed due to poor coupling of the mouthguard with the upper jaw during impact from deliberate removal or loose fitting. With a professionally built custom mouthguard, this possibility of improper coupling is lower as the device “snap fits” onto the teeth.

### Implications for Head Injury Research

On the collegiate football field, we found that nonimpacts could account for most recordings on a small light-weight wearable head impact sensor. Typically, for an offensive lineman involved in most plays over a game, we can expect the instrumented mouthguard to record up to 50 head impacts and about 1000 nonimpacts (that are mostly off-teeth recordings). It is important in this context that classifiers be evaluated for sensitivity and precision, instead of sensitivity and specificity, since even 90% specificity would lead to 100 false positive detections, which is twice the number of actual head impacts. Given over 90% sensitivity and precision, up to 5 head impacts may be missed and 5 detected head impacts may be false positives. To collect a dataset for studying subconcussive head impacts, this level of sensitivity should be sufficient to allow for gathering of a large amount of data in a relatively short amount of time, since 90% of the helmet contacts can be captured. However, to gather concussion data, which are rare occurrences, higher sensitivity may be desirable. Future wearable head impact sensor designs should focus on (1) optimizing skull coupling and minimizing noise in the head impacts, such that these events may be more clearly distinguished from nonimpacts, and (2) ensuring player compliance such that the device is worn and well-coupled during all potential contact activities.

## Conclusion

In summary, we developed a head impact classifier trained on field data from collegiate football. In the future, this classifier may be incorporated into an instrumented mouthguard and validated for real-time head impact detection on the field with few false positives and false negatives. Such a validated system can help gather high quality human head impact data from the field for traumatic brain injury research. Similar methodology may also be adapted to other applications that need to classify human activities using wearable inertial sensors.

## Electronic supplementary material

Supplementary Information

Supplementary Dataset 1
