# Supplementary material for: Detection of American Football Head Impacts Using Biomechanical Features and Support Vector Machine Classification
Source: Sci Rep. 2017 Dec 21;8:855. doi: 10.1038/s41598-017-17864-3 (PMC5762632; doi:10.1038/s41598-017-17864-3)
Supplement: Supplementary file 1 — Supplementary Information [file 41598_2017_17864_MOESM1_ESM.doc]

**Supplementary Figures and Table for Manuscript Titled:**

Detection of American Football Head Impacts Using Biomechanical Features and Support Vector Machine Classification

# **Authors:**

# Lyndia C. Wu^1^, Calvin Kuo^1^, Jesus Loza^1^, Mehmet Kurt^2^, Kaveh Laksari^3^, Livia Z. Yanez^1^, Daniel Senif^1^, Scott C. Anderson^1^, Logan E. Miller^4^, Jillian E. Urban^4^, Joel D. Stitzel^4^, David B. Camarillo^1^

# ^1^Stanford University, Stanford, CA, USA

# ^2^Stevens Institute of Technology, Hoboken, NJ, USA ^3^University of Arizona, Tucson, AZ, USA

# ^4^Wake Forest University, Winston-Salem, NC, USA


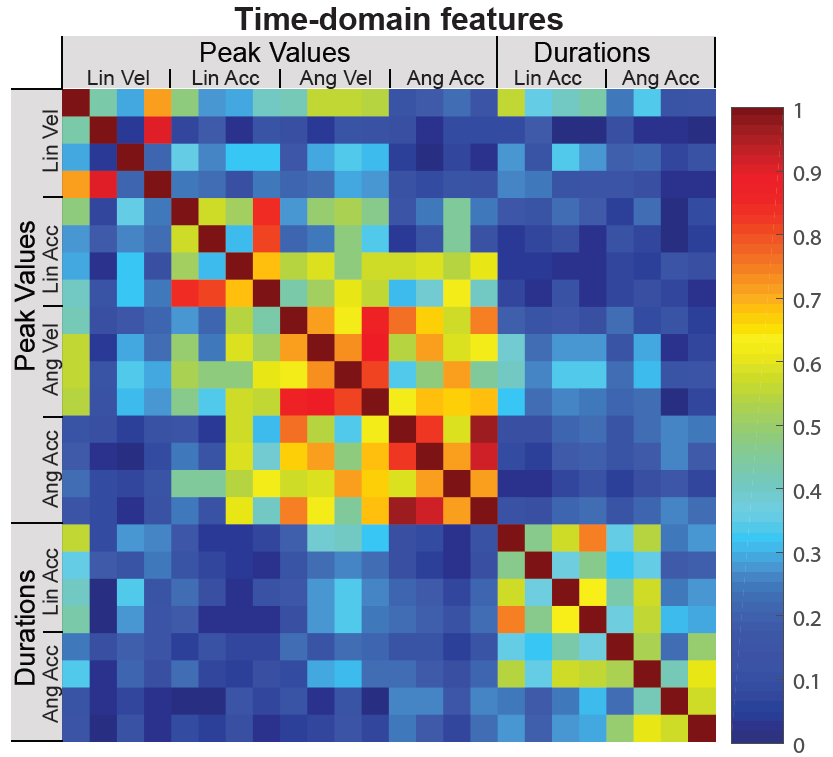


**Supplemental Fig. 1 – Pearson’s Correlation Coefficients Among Time Domain Features**. Angular peak kinematics features tended to have higher correlations between each other.


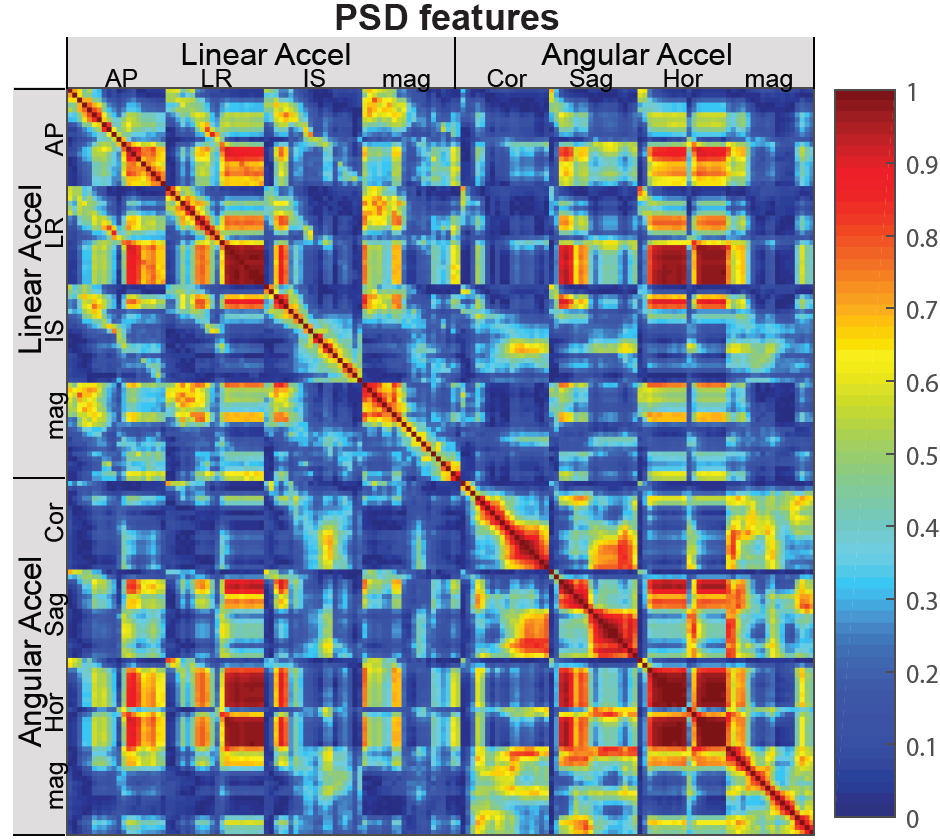
 **Supplemental Fig. 2 – Pearson’s Correlation Coefficients Among PSD Features**. The AP and LR linear acceleration features at neighboring frequencies tend to be highly correlated with each other, and these were also highly correlated with angular velocity PSD features in the sagittal and horizontal planes.


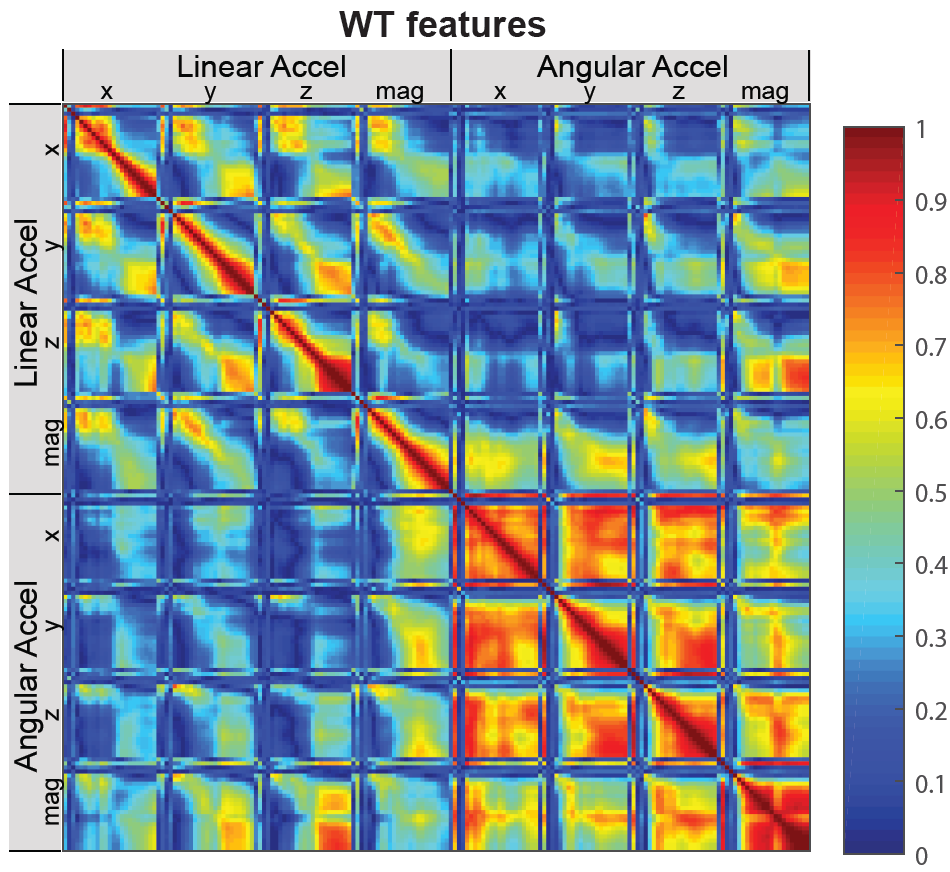


**Supplemental Fig. 3 – Pearson’s Correlation Coefficients Among WT Features**. The angular acceleration WT features tend to be highly correlated with each other.


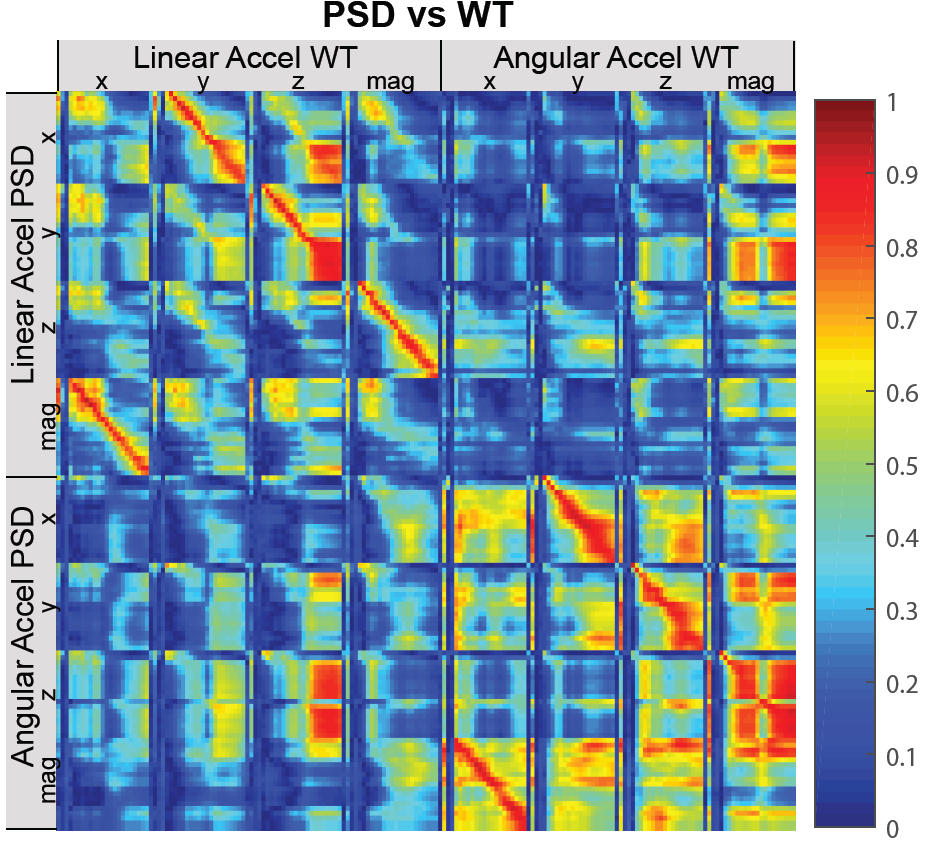


**Supplemental Fig. 4 – Pearson’s Correlation Coefficients Between PSD and WT Features**. The linear acceleration PSD features had high correlation coefficients with linear acceleration WT features, and similar observations were made for the angular PSD and WT features.


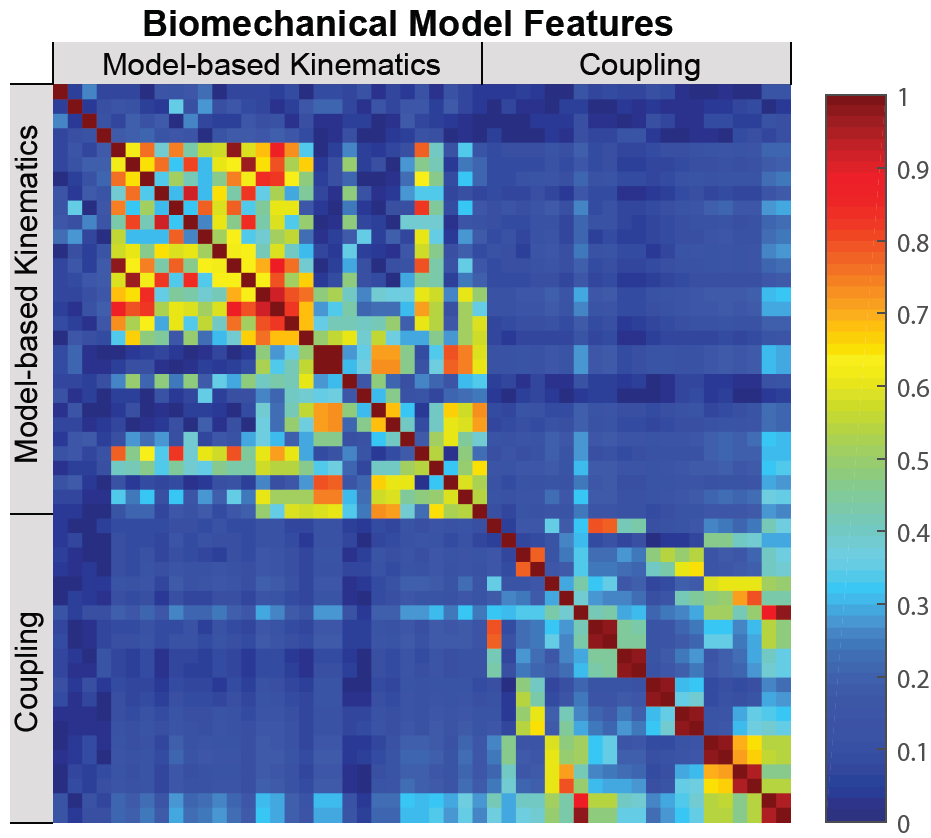


**Supplemental Fig. 5 – Pearson’s Correlation Coefficients Among Biomechanical Model Features**. The model-based kinematics features and some coupling features tend to be correlated with each other.

**Supplemental Table – All Features**

| **Feature Number** | **Feature Descriptions** |
| --- | --- |
| 1-4 | Peak change in linear velocity (anterior-posterior, left-right, inferior-superior, vector magnitude) |
| 5-8 | Peak absolute linear acceleration (anterior-posterior, left-right, inferior-superior, vector magnitude) |
| 9-12 | Peak change in angular velocity (coronal, sagittal, horizontal, vector magnitude) |
| 13-16 | Peak absolute angular acceleration (coronal, sagittal, horizontal, vector magnitude) |
| 17-20 | Duration of linear acceleration impulse (anterior-posterior, left-right, inferior-superior, vector magnitude) |
| 21-24 | Duration of angular acceleration impulse (anterior-posterior, left-right, inferior-superior, vector magnitude) |
| 25-44 | Anterior-posterior linear acceleration PSD at 10Hz, 20Hz, …, 200Hz |
| 45-64 | Left-right linear acceleration PSD at 10Hz, 20Hz, …, 200Hz |
| 65-84 | Inferior-superior linear acceleration PSD at 10Hz, 20Hz, …, 200Hz |
| 85-104 | Linear acceleration vector magnitude PSD at 10Hz, 20Hz, …, 200Hz |
| 105-122 | Coronal plane angular acceleration PSD at 10Hz, 20Hz, …, 180Hz |
| 123-140 | Sagittal plane angular acceleration PSD at 10Hz, 20Hz, …, 180Hz |
| 141-158 | Horizontal plane angular acceleration PSD at 10Hz, 20Hz, …, 180Hz |
| 159-176 | Angular acceleration vector magnitude PSD at 10Hz, 20Hz, …, 180Hz |
| 177-196 | Peak anterior-posterior linear acceleration WT over time at 10Hz, 20Hz, …, 200Hz |
| 197-216 | Peak left-right linear acceleration WT over time at 10Hz, 20Hz, …, 200Hz |
| 217-236 | Peak inferior-superior linear acceleration WT over time at 10Hz, 20Hz, …, 200Hz |
| 237-256 | Peak linear acceleration vector magnitude WT over time at 10Hz, 20Hz, …, 200Hz |
| 257-274 | Peak coronal plane angular acceleration WT over time at 10Hz, 20Hz, …, 180Hz |
| 275-292 | Peak sagittal plane angular acceleration WT over time at 10Hz, 20Hz, …, 180Hz |
| 293-310 | Peak horizontal plane angular acceleration WT over time at 10Hz, 20Hz, …, 180Hz |
| 311-328 | Peak angular acceleration vector magnitude WT over time at 10Hz, 20Hz, …, 180Hz |
| 329-360 | For WT of each component and vector magnitude of linear acceleration and WT of each component and magnitude of angular acceleration, there were four features: the frequency of the peak WT amplitude, value of the peak WT amplitude, number of milliseconds of the peak WT amplitude relative to the time-domain peak, and bandwidth of the WT transform (the frequency at which the peak WT amplitude dropped below 10% of the maximum value). |
| 361-364 | These features determine if there is a sign match between predicted coordinates in the biomechanical model. The matching signs represent an assumption that the human head and neck typically does not inflect. E.g., if the neck is flexed anteriorly, the head is also flexed anteriorly and not extended posteriorly. |
| 365-372 | Estimated displacement and rotation of the neck and torso using the biomechanical model. The displacement of the neck is defined as the displacement of the point connecting the head and neck. The displacement of the torso is defined as the displacement of the point connecting the neck and torso. The rotation of the neck and torso are defined with respect to their joint angles as defined in the biomechanical model. |
| 373-390 | Estimate translational and rotational velocity and acceleration of the torso and neck using the biomechanical model. The velocity and acceleration of the torso is defined as the velocity and acceleration of the point connecting the neck and torso. The velocity and acceleration of the neck is defined as the velocity and acceleration of the point connecting the neck to the head. Rotational velocities and accelerations were defined as the velocities and accelerations of the respective joint angles. |
| 391-411 | Ratios between linear acceleration and angular acceleration in magnitude and components. These features represent an implicit assumption that the head and neck can be represented roughly as a mechanical inverted pendulum, with the pendulum length (representing the neck length) as the ratio between the linear acceleration and angular acceleration. Ratios were determined at each sample in a single impact, and then the maximum was taken to obtain the peak features, averaged to obtain mean features (for head impacts, approximately neck length). Standard deviations over time were also obtained (for head impacts, neck length doesn’t change over time). |
